# Why choose pediatrics? A survey on factors influencing Chinese high school students’ choice of pediatrics major

**DOI:** 10.3389/fmed.2025.1646958

**Published:** 2026-01-21

**Authors:** Wanxin Liu, Xiaoqiang Wang, Kai Zhu, Qiugen Wang, Zhi Long, Chencong Lv, Chaoqun Yang, Guoying Deng, Qian Wang

**Affiliations:** 1 Yueyang Hospital of Integrated Traditional Chinese and Western Medicine, Shanghai University of Traditional Chinese Medicine, Shanghai, China; 2 Shanghai General Hospital, Shanghai Jiao Tong University School of Medicine, Shanghai, China; 3 Shanghai Jiao Tong University School of Medicine, Shanghai, China; 4 Sun Yat-sen Memorial Hospital, Sun Yat-sen University, Guangzhou, China

**Keywords:** pediatrics, career intention, high school students, medical specialty choice, healthcare workforce

## Abstract

**Objectives:**

To understand factors associated with senior high school students’ willingness to apply for pediatrics in order to inform recruitment strategies.

**Methods:**

An anonymous questionnaire was distributed to 9,574 high school students. The questions covered the following topics: students’ intention to apply for pediatrics, personal and family information, factors impacting a student’s choice of major, and cognizance of the state of pediatrics (including education and working conditions). univariate and multivariate logistic regression analysis and chi-square tests were used to compare students with and without a clear pediatric intention and to explore influencing factors.

**Results:**

Only 6.4% (513/8,050) of senior high school students had intention to apply for pediatrics (IAP). The goal was directly tied to private and family information. Students’ IAP is favorably connected with their interests and well-defined career objectives, particularly when considering COVID-19. The way that students view pediatric illnesses has an impact on their willingness as well. Students’ significant decisions are influenced by their understanding of pediatrics; the more positive their perceptions are of the remuneration and working circumstances for physicians, the more probable it is that they will have an IAP.

**Conclusion:**

This survey reveals the hesitancy of high school students in selecting pediatrics as their career path and explores potential contributing factors. Furthermore, these findings offer insights into the development of healthcare services in emerging economies.

## 1 Introduction

The availability of an adequate pediatric workforce is critical to safeguarding children’s health and has far-reaching implications for various key indicators (1). With approximately 279 million individuals under the age of 18, China accounts for nearly 15% of the global child population (2). However, the country is facing a critical shortage of pediatricians. In 2017, there were only around 100,000 pediatricians serving roughly 260 million children aged 0–14, that this is a ratio of roughly 0.38 pediatricians per 1,000 children (3). Although the number increased to approximately 150,000 by the end of 2021, it still falls short of the government’s target of 174,834 pediatricians, or 0.69 per 1,000 children, as set out in the 2016 *Opinions on Strengthening the Reform and Development of Children’s Medical and Health Services*. Compounding the issue, a national survey reported a turnover rate of 12.6% among pediatric physicians between 2017 and 2019 (4).

This persistent shortfall has serious implications for healthcare access, service quality, and child health outcomes. Delays in treatment, increased physician workload, and reduced continuity of care are just some of the consequences. In response, the Ministry of Education recently upgraded pediatrics from a secondary specialty to an independent undergraduate major, aiming to improve early recruitment and expand the professional pipeline (5, 6). This policy change happened in 2016 and it means now pediatrics can be chosen as an undergraduate major.

In China’s higher education system, students typically decide whether to pursue medicine before entering university. They first receive general medical training at the undergraduate level, followed by specialization during postgraduate education. Despite increasing attention to pediatric education, existing research has mainly focused on doctor–patient relationships (7), medical student competencies (8), and workforce retention. However, research into attracting students into pediatrics is still in its early stages. In China, students begin to differentiate their career interests in high school. The willingness of senior high school students to pursue pediatrics directly impacts the stability of medical school enrollment in that specialty and reflects society’s recognition of the profession.

Therefore, this study surveyed senior high school students to assess their willingness to apply for a pediatrics major and to explore the factors influencing that willingness. Understanding these factors is crucial for developing effective recruitment strategies and ultimately mitigating the pediatrician shortage at its source.

## 2 Materials and methods

### 2.1 Participants

This study was conducted between 2021 and 2023. Using a cluster sampling method, we recruited 9,574 senior high school students (age 16–20) from four provinces selected to represent China’s geographic and socioeconomic diversity: Guangdong (eastern/coastal, more economically developed), Anhui and Henan (central provinces with mid-range development), and Sichuan (western region). This regional spread enhances the representativeness of the sample. Academic status comprised first-year (Senior 1/Grade 10), second-year (Senior 2/Grade 11), third-year (Senior 3/Grade 12), and repeat class students (post–Senior 3 students preparing for a second attempt at the national entrance examination). For analytic consistency, we defined the graduating cohort as the combination of third-year and repeat class students, reflecting their shared proximity to the college entrance examination and comparable decision context. The distribution across the three analytic categories was 36.9% first-year, 43.6% second-year, and 19.5% graduating cohort. Students were classified according to their subject-track selection under the mainstream or reformed gaokao system. The science track denotes combinations centered on physics, chemistry, and biology; the humanities track denotes combinations centered on history, geography, and politics. In some provinces implementing curriculum reforms, certain subject combinations may allow cross-track eligibility for medical majors; we therefore analyze track as a self-reported academic orientation while noting potential policy heterogeneity across provinces.

All subjects met the following criteria: (1) they were informed about and agreed to participate in the study; (2) They can correctly understand the questionnaire content; (3) they had the intention to apply to a university or college. This study was approved by the Ethics Committee of the First People’s Hospital of Shanghai (Approval No.2019KY058).

### 2.2 Questionnaire

The survey method used was a written questionnaire. The initial questionnaire was designed by reviewing the literature and conducting expert interviews. A pre-survey was conducted online with 200 senior high school students from four provinces: Anhui, Guangdong, Sichuan, and Henan. The final questionnaire was then developed based on the results of the pre-survey, with a Cronbach’s alpha coefficient of 0.764, meeting the requirements for general exploratory experiments. The final questionnaire consisted of 28 items divided into three sections: (1) Demographic and family background (gender, grade, academic performance tier, course track, family income, parents’ occupations, etc.), (2) Factors influencing career choice (personal interest in medicine, medical volunteer experience, sources of information on majors, career planning status, importance of job stability/benefits, impact of COVID-19 on career choice), and (3) Understanding and perceptions of pediatric medicine (views on the nature of a pediatrician’s work, perceived alignment with reality, career prospects, training time required, work environment, doctor-patient relationship, public image, salary and benefits, etc.).

Questionnaires were administered offline via cluster sampling (random selection of classes within each grade; all students in selected classes were invited). Responses were completed anonymously and independently; questionnaires with logic errors or missing key fields were excluded prior to analysis. Students rated their willingness to apply to a pediatrics major on a five-point Likert scale (1 = extremely weak, 5 = extremely strong). We dichotomized this for analysis, with ratings of 4 or 5 considered a strong intention to pursue pediatrics (IAP = yes).

### 2.3 Statistical analysis

The collected questionnaires were uniformly entered into Excel software, and the entered data were analyzed using SPSS 21.0 software. For analysis, we dichotomized this item: IAP = 1 for ratings 4–5 (strong intention) and IAP = 0 for ratings 1–3 (not strong/undecided). All binary predictors were dummy-coded with Yes = 1 and No = 0 (reference); multi-category predictors were dummy-coded with reference categories specified in the tables. Frequency and percentage were calculated as descriptive statistics. As categorical variables, students’ socio-demographic details and family background were analyzed with the chi-square test. To examine factors associated with intention to apply for pediatrics, univariate logistic regression was first performed on each factor (Tables 2, 3 show these ORs). Then a multivariate logistic regression model was constructed including all variables (or all significant variables) to identify independent predictors of IAP. Logistic regression results are reported as odds ratios (ORs) with 95% confidence intervals (CIs) and two-sided *p*-values (to three decimals when ≥ 0.001; otherwise *p* < 0.001). Statistical significance was set at α = 0.05 (two-sided).

## 3 Results

### 3.1 Students’ willingness

A total of 9,574 questionnaires were distributed, and after excluding those with logical errors and incomplete questionnaires, 8,050 valid questionnaires were collected, with a valid response rate of 84.1%. For interpretability, we note that “strong intention to apply for pediatrics” refers to students selecting 4 or 5 on the five-point willingness scale (coded as IAP = 1); all other responses were coded as IAP = 0.

Only 6.4% of surveyed high school students had a strong intention to apply for a pediatrics major. In other words, roughly 1 in 16 students showed solid interest in pediatrics, highlighting the pipeline challenge. The differences in demographic information, personal experiences, medical knowledge, and career awareness among the survey participants were statistically significant (*p* < 0.05).

### 3.2 Demographic information

The demographics of the research sample are presented in Table 1. All items were completed. Among the surveyed individuals, males and females accounted for 44.2% and 55.9%, respectively. Only 5.4% of male students and 7.1% of female students expressed an intention to pursue pediatrics as their major. The distribution of students across grade levels was as follows: 36.9% in senior one, 43.6% in senior two, and 19.5% in the graduating cohort. 7.6% of senior one students were interested in pursuing pediatrics (224/2,973), while the proportion for senior two students was 5.8% (202/3,508), and for graduating cohort students, it was 5.5% (87/1,570). By academic performance, 8.0% (108/1,345) of students in the bottom tier reported a strong intention to apply for pediatrics, higher than in the middle and top tiers.

**TABLE 1:** Influence of demographic factors on senior high school students’ willingness to major in pediatrics.

Item	*N* = total	students with an IAPS [*n* (%)]		χ^2^	*P*-value
Gender				13.985	<0.001
Male	3,558	192	5.40%		
Female	4,492	321	7.15%		
Performance ranking				22.083	0.003
Top 30%	2,985	150	5.03%		
30%–70%	3,720	255	6.85%		
Bottom 30%	1,345	108	8.03%		
Grade					<0.001
Senior one	2,973	224	7.53%		
Senior two	3,508	202	5.76%		
Graduating cohort	1,570	87	5.54%		
Your selection of courses				14.361	
Humanities	2,292	197	8.60%		
Science	5,245	316	6.02%		
Family income (dollars)/month				18.286	0.019
<300	686	38	5.54%		
≥300 < 700	3,228	217	6.72%		
≥700 < 1,400	2,871	164	5.71%		
≥1,400 < 2,800	1,068	55	5.15%		
≥2,800	197	19	9.64%		
Parents are medical workers or not				20.097	0.003
Father	139	5	3.60%		
Mother	138	4	2.90%		
Both	265	22	8.30%		
None	7,508	482	6.42%		
Location of hometown				28.014	<0.001
Tier-1 city	62	3	4.84%		
Tier-2 city	123	4	3.25%		
Tier-3 city	753	59	7.84%		
Towns	5,046	482	9.55%		
Rural	2,066	124	6.00%		
The attitude of family members or acquaintances towards pursuing a career in pediatrics.				612.012	<0.001
Strongly oppose	72	3	4.17%		
Oppose	442	15	3.39%		
Neutral	5,620	184	3.27%		
Support	1,831	286	15.62%		
Strongly support	85	25	29.41%		
Who influenced you the most in your choice of college major				34.475	<0.001
Myself	4,830	327	6.77%		
Parents	1,552	95	6.12%		
Teachers	553	31	5.61%		
Relevant professionals	1,115	60	5.38%		

IAP, Intention to Apply for Pediatrics. IAP = 1 if willingness rating 4–5 on a five-point scale; otherwise IAP = 0. Hometown location categories:Tier-1\2\3 city: It is divided according to the position and leading role of cities in national or global political, economic and other social activities and radiation driving ability; Tier-1/2, major metropolitan cities; Tier-3, lower-tier prefecture-level cities; Towns = county-level town seats/small cities; Rural = villages. Academic performance were self-reported relative rank, defined within school and within grade as top 30%, middle 40%, and bottom 30%. Selection of Courses: Humanities vs. science based on China’s college entrance exam requirements.

By course track, 8.5% of humanities-track students and 6.0% of science-track students reported strong intention to apply for pediatrics (197/2,292 vs. 316/5,245). Given provincial differences in subject-combination rules under curriculum reforms, this contrast should be interpreted with caution.

Family information includes four aspects: income, parental occupations, family location, and the family’s attitude toward pediatrics. Students with a family income of ≥2,800 dollars/month showed a higher willingness, at 9.64% (19/197). A significant difference was observed between students with both parents working in medical institutions and those whose parents did not. Students with parents working in healthcare professions showed a significantly higher inclination toward pursuing pediatrics, with a proportion of 8.3% (22/265), which was much higher than those whose parents did not (6.42%, 482/7,508; *p* = 0.03). We note the small n for the medical-parent subgroup (265 students), so this difference should be interpreted with caution. Family attitude exhibited the largest gradient: 16.23% (311/1,916) of students whose families supported a pediatrics major expressed strong intention, versus 3.27% (184/5,620) with neutral attitudes and 3.50% (18/514) with negative attitudes—nearly five times higher in the supportive group. 9.55% of students from towns (482/5,046) expressed strong intention to apply for pediatrics, compared with 7.84% of those from Tier-3 cities (59/753) and 6.00% from rural areas (124/2,066). Tier-1 and Tier-2 cities showed the lowest proportions (4.84%, 3/62; 3.25%, 4/123).

### 3.3 Factors influencing career choices

Factors influencing students’ career choices are presented in Table 2. Univariate analysis shows a significant positive correlation between students’ interest in pediatrics and IAP (adjusted OR = 4.053, *p* < 0.001). Although only 7.2% (585/8050) of students had a strong interest in pediatrics, 42.6% (249/585) of them express an intention to pursue pediatrics. High school students who had participated in medical practice or science activities showed higher interest in pediatrics (9.11% vs. 5.35%, adjusted OR = 1.354, *p* < 0.001). Similarly, those who reported having a clear career plan were more likely to intend to apply for pediatrics (7.58% vs. 5.50%, OR = 1.408, *p* < 0.001), but the association was not statistically significant in the multivariable logistic regression. The clear professional direction effect attenuated after adjustment—likely reflecting shared variance with family support and intrinsic interest in medicine—suggesting that planning per se may operate through these proximal motivations.

**TABLE 2:** Factors influencing career choices.

Item	*N* = total	Students with IAPS [*n*(%)]		Univariate regression		Multivariate regression	
				*P*-value	OR (95% CI)	*P*-value	OR (95% CI)
Interest in pediatrics				*P* < 0.001	4.729 (4.136–5.408)	*P* < 0.001	4.053 (3.532–4.651)
Totally uninterested	1,392	22	1.58%				
Uninterested	2,365	50	2.11%				
Neutral	3,708	192	5.18%				
Interested	507	213	42.01%				
Very interested	78	36	46.15%				
Participated in medical practice or popular science				<0.001	1.775 (1.476–2.135)	0.005	1.354 (1.098–1.654)
Yes	2,195	200	9.11%				
No	5,855	313	5.35%				
How to find out about college majors						0.001	
Traditional media	1,855	57	3.07%	<0.001			
New media	2,381	128	5.38%	<0.001	1.792 (1.304–2.464)	0.003	1.67 (1.191–2.342)
University advocacy	2,668	209	7.83%	<0.001	2.681 (1.988–3.616)	*P* < 0.001	1.88 (1.366–2.587)
Surrounding experience	990	99	10.00%	<0.001	3.505 (2.506–4.903)	0.001	1.865 (1.2871–2.703)
Unawareness	156	20	12.82%	<0.001	4.639 (2.708–7.947)	0.001	2.864 (1.531–5.361)
Career in line with interests is				0.389	1.053 (0.936–1.186)		
Very important	2,973	168	5.65%				
Important	3,957	276	6.97%				
Average importance	922	60	6.51%				
less important	176	9	5.11%				
Very unimportant.	22	0	0.00%				
Having a clear professional direction or career plan				<0.001	1.408 (1.178–1.685)	0.111	1.176 (0.964–1.434)
Agree	3,363	255	7.58%				
Disagree	4,687	258	5.50%				
Good job stability and benefits of a future career is				<0.014	1.162 (1.031–1.309)	0.013	1.180 (1.036–1.344)
Very important	2,454	196	7.99%				
Important	4,027	208	5.17%				
Average importance	1,331	103	7.74%				
less important	203	6	2.96%				
Very unimportant.	35	0	0.00%				
Anxiety level during the Covid-19				<0.001	1.224 (1.113–1.345)	0.017	1.138 (1.023–1.266)
Very low	3,344	117	3.50%				
Low	3,129	207	6.62%				
Average	1,258	97	7.71%				
High	213	21	9.86%				
Very high	106	11	10.38%				
Deepened recognition of the pediatric medicine landscape post-COVID				<0.001	4.109 (3.425–4.929)	P < 0.001	2.362 (1.934–2.885)
Yes	1768	260	14.71%				
No	6281	253	4.03%				

Compared to traditional media (newspapers, TV) as a source of career information, those who learned about majors through university outreach or personal experiences were roughly 1.7–2.0 times more likely to consider pediatrics (*p* < 0.01), according to our logistic regression.

A majority of students, 80.5%, prioritize job stability, good benefits, and comprehensive insurance for their future careers. Greater emphasis on job stability/benefits was associated with higher odds after adjustment (adjusted OR = 1.180; *p* = 0.013; univariate OR = 1.162, *p* = 0.014) The COVID-19 pandemic has also influenced the professional choices of high school students. A small subset of students (3.9%) reported feeling panic about career choices during COVID-19, and this group had a higher rate of interest in pediatrics (10.38% vs. about 6.37% in the overall sample, adjusted OR = 1.138, *p* = 0.01). This effect was modest relative to post-COVID recognition. Those who reported deeper recognition of pediatrics after COVID-19 showed 14.71% IAP (260/1,768) versus 4.03% among others (253/6,281), corresponding to a strong association (univariate OR = 4.109, *p* < 0.001; adjusted OR = 2.362, *p* < 0.001).

### 3.4 Students’ comprehension of pediatric medicine

As shown in Table 3, students were asked about various aspects of a pediatric career: the perceived essence of a pediatrician’s work (altruistic service vs. technical or profit-driven), whether they think a pediatric career matches their expectations of reality, the career prospects and time investment required, the current job market situation for pediatricians, and qualitative aspects like work pressure, work environment, social status, sense of professional achievement, doctor-patient relationships, public image, and salary/benefits.

**TABLE 3:** Students’ comprehension of pediatric medicine.

Item	*N* = total	Students with IAPS [*n*(%)]		Univariate regression		Multivariate regression	
				*P*-value	OR (95% CI)	*P*-value	OR (95% CI)
Which phrase characterizes a pediatrician						0.252	–
Interests are paramount	147	9	6.1%	0.838	0.93		
Service	3,007	172	5.7%	0.436	1.318		
Skilled labor	1,870	148	7.9%	0.983	0.993		
Helping others	3,026	184	6.1%	0.019			
Perceptions of pediatrics match reality				<0.001	1.277 (1.162–1.402)	<0.001	1.285 (1.168–1.415)
Strongly agree	406	58	1.6%				
Agree	1,824	165	2.1%				
Neutral	3,182	135	5.2%				
Disagree	2,247	110	42.0%				
Strongly disagree	391	45	46.2%				
Awareness of future directions for pediatric graduates				<0.001	2.182 (1.961–2.428)	<0.001	1.537 (1.354–1.744)
Strongly disagree	1,418	36	2.5%				
Disagree	2,336	78	3.3%				
Neutral	3,615	264	7.3%				
Agree	584	110	18.8%				
Strongly agree	97	25	25.8%				
Know the time cost of becoming a pediatrician				<0.001	1.826 (1.656–2.012)	<0.001	1.235 (1.098–1.389)
Strongly disagree	2,119	59	2.8%				
Disagree	2,523	138	5.5%				
Neutral	2,947	227	7.7%				
Agree	373	66	17.7%				
Strongly agree	88	23	26.1%				
The current pediatric employment situation				<0.001	2.037 (1.840–2.256)	0.002	1.303 (1.105–1.537)
Very bad	1,229	22	2.8%				
Bad	1,909	78	5.5%				
Neutral	3,478	198	7.7%				
Well	1,294	186	17.7%				
Very well	140	29	26.1%				
Work pressure of pediatricians				<0.001	1.709 (1.553–1.881)	0.078	
Very low	1,112	19	1.7%				
Low	1,724	66	3.8%				
Average	3,495	236	6.8%				
High	1,484	163	11.0%				
Very high	235	29	12.3%				
The social status of pediatricians				<0.001	1.858 (1.684–2.050)	0.024	1.236 (1.029–1.486)
Very low	997	15	1.5%				
Low	1,632	67	3.8%				
Average	3,439	189	6.8%				
High	1,716	198	11.0%				
Very high	266	44	12.3%				
Pediatrician career fulfillment				<0.001	1.795 (1.635–1.972)	0.040	1.221 (1.009–1.477)
Very low	1,071	17	1.6%				
Low	1,397	51	3.7%				
Average	3,430	196	5.7%				
High	1,743	188	10.8%				
Very high	409	61	14.9%				
The work environment of pediatricians				<0.001	1.929 (1.747–2.130)	0.072	
Very bad	1,171	20	1.7%				
Bad	1,732	64	3.7%				
Neutral	3,561	212	6.0%				
Well	1,381	187	13.5%				
Very well	205	30	14.6%				
Cognition of doctor-patient relationship				<0.001	0.803 (0.718–0.898)	0.003	0.841 (0.748–0.944)
Very intense	592	42	7.1%				
Intense	3,810	285	7.5%				
Neutral	2,606	137	5.3%				
Harmonious	984	47	4.8%				
Very harmonious	58	2	3.4%				
The public image of the pediatrician				<0.001	1.723 (1.571–1.891)	0.002	1.292 (1.098–1.521)
Very bad	865	17	2.0%				
Bad	1,369	55	4.0%				
Neutral	3,223	167	5.2%				
Well	2,037	184	9.0%				
Very well	556	90	16.2%				
Pediatrician’s salary and treatment				<0.001	1.594 (1.449–1.752)	<0.001	1.515 (1.272–1.805)
Very poor	1,078	24	2.2%				
Poor	1,627	64	3.9%				
Neutral	3,421	234	6.8%				
Well	1,682	157	9.3%				
Very well	242	34	14.0%				

Students overwhelmingly view a doctor’s work as helping or serving others (over 70% selected these altruistic motives), regardless of single-factor or multi-factor regression, students’ views on the essence of a doctor’s work do not correlate with IAPS, this idealistic perception did not significantly differentiate those interested in pediatrics vs. those not.

A total of 27.7% of high school students believe that a pediatric career aligns with reality, and students who believed that the pediatric career ideal matches reality were more likely to be interested (aOR = 1.285, <0.001). Only 8.5% of students reported knowing the career prospects of pediatrics and 5.7% knew the length of training. In adjusted analyses, awareness of career prospects (aOR = 1.537, *p* < 0.001) and awareness of the time cost (aOR = 1.235, *p* < 0.001) were both associated with higher odds of intending to pursue pediatrics. Awareness of the realities of the profession was generally low among students; however, those few who were informed about pediatric career prospects or understood the lengthy training required were about three times more likely to choose pediatrics. This suggests that better informed students might self-select into the field (or conversely, that those seriously considering the field sought out information).

That is 36% of 8,050 vs. 18% who think pediatricians’ working environment is not poor, and a majority perceived high workload/stress. Students holding a positive view of the work environment had higher odds of strong interest in univariate analysis (OR = 1.929, *p* < 0.001), although this association was not significant in the adjusted model. A total of 23% of students believed pediatricians enjoy high social status, and 27% perceived high professional achievement in pediatrics. Students endorsing these views were more likely to express strong intention to choose pediatrics (univariate ORs≈1.8 for both). In adjusted analyses, associations remained significant though smaller—social status: aOR = 1.236 (95% CI 1.029–1.486); career fulfillment/achievement: aOR = 1.221 (95% CI 1.009–1.477). Only 12.9% of students believed the pediatric doctor–patient relationship is harmonious; most were neutral or viewed it as tense.

A majority of students (5,816/8,058, 72.2%) reported a positive public image of pediatricians. Those with a positive image were more likely to express strong intention to choose pediatrics (univariate OR = 1.723, *p* < 0.001), and the association persisted in multivariable analysis (aOR = 1.292, 95% CI 1.098–1.521, *p* = 0.002). Only 23.9% (242/8,050) of students rated pediatricians’ income/benefits as very well; most viewed it as poor or average. A total of 14.0% of students who believed compensation is good reported strong intention to choose pediatrics, versus 3% among those who believed it is poor or very poor. Perceiving better compensation was associated with higher odds of intending to choose pediatrics in the multivariable model (adjusted OR = 1.515, *p* < 0.001).

## 4 Discussion

This survey of over 8,000 high school students found that only about 6% are strongly interested in a pediatrics career. Female students, those with family support or medical family background, and those with personal interest and relevant experience were more likely to consider pediatrics. Students who perceived better career prospects, higher status, and better remuneration in pediatrics also showed greater interest. In contrast, many students held concerns about the field – such as high workloads, low pay, and strained doctor-patient relationships – which likely dissuade them from pediatrics.

Our survey indicates that senior high school students already perceive pediatrics as difficult and under-rewarded: only 12.9% believed the pediatric doctor–patient relationship is harmonious (1,042/8,050), and a majority rated the work environment as poor, viewed workload and stress as high, and considered compensation to be low. These views are broadly consistent with prior research describing high medical risk, heavy workload, lower income, and limited promotion opportunities for pediatricians in China (9–14). It is therefore plausible that awareness or sensitivity to these challenges suppresses interest in the field, contributing to the shortage pipeline we document.

In China’s diverse family structures [from nuclear to intergenerational households (15)] and in a society with declining birth rates, pediatricians often interact with not just parents but entire families across generations. They find themselves not only treating the child but also managing the family’s anxieties. Combined with a group practice setting, this makes pediatricians more susceptible to conflicts in doctor-patient interactions. These features align with students’ perceptions of strained relationships and demanding working conditions and are echoed by reports of rising medical disputes over the past two decades (12, 16). Clinical risk is further amplified because pediatric diseases may progress rapidly and because there are fewer pediatric-specific tests, treatments, and medicines, placing a premium on clinical judgment. At the same time, the economic valuation of pediatric work has lagged—pediatricians in comprehensive hospitals often report lower salaries than peers in other specialties (14, 17)—and heavy service demands can constrain time for professional development, further training, and research, impeding career advancement(18–20). Students’ perceptions in our survey—poor environment, strained relationships, low pay—map onto these realities and likely reduce perceived attractiveness of pediatrics.

The cultivation of talent plays a pivotal role in ensuring the future supply of medical professionals and fostering industry advancement. Student enrollment serves as the fundamental basis for nurturing future medical practitioners. In response to the significant shortage of pediatricians, starting from 2016, the Chinese government re-introduced certain undersupplied professions such as pediatrics into undergraduate education (in China, medical students typically select their specialty during postgraduate training). Therefore, it is imperative to investigate their current enrollment status and identify factors that may influence students’ willingness to pursue this field. The findings of this study reveal a limited inclination among senior high school students in China towards specializing in pediatrics, which aligns with the declining number of applicants for pediatric programs in medical schools nationwide.

There were statistically significant differences in enrollment willingness among senior high school students of different genders, academic performance, grades, family information and various experiential and perceptual factors. Senior high school students who had career plans received support from both their families and acquaintances for enrollment, while also benefiting from increased exposure to the pediatric profession through pandemic-related publicity and experiences. Consequently, this group exhibited a higher proportion of strong enrollment willingness (11, 21).

Consistent with prior work, gender is associated with specialty intentions among high-school students (22, 23). In our sample, female students were more likely to express strong intention to choose pediatrics than male students (≈7% vs. ≈5%; aOR = 1.367). This pattern likely reflects differential sorting by interests and incentives rather than ability per se: pediatrics involves intensive communication and caregiving with children and families, domains in which many students (often women) report stronger identification and patience (24). At the same time, pediatrics is perceived to offer lower pay and slower advancement and to make technical expertise less visible relative to some procedure-heavy fields (25, 26); consequently, some male students may prioritize specialties they perceive as better compensated or more prestigious (27). In response to workforce shortages, admission and entry policies have at times emphasized supply expansion in pediatrics. The fact that entry requirements for pediatrics have been lowered (28) to attract more applicants may inadvertently label it as a “less competitive” field, potentially influencing high-achieving students’ interest.

We observed that senior one students were more likely than the graduating cohort to express strong intention to pursue pediatrics, a pattern consistent with career-development theory (10),whereby students’ choices often shift from more idealistic to more pragmatic as age and career maturity increase(10, 29, 30). In fact, students reporting a clearer career plan were more inclined to consider pediatrics; although this association attenuated after adjustment, the direction remained consistent, suggesting that students with clear plans being more rational and less swayed by negatives. This finding underscores the value of early career planning support – students who have thought through their goals are less deterred by challenges of a profession.

Importantly, structured career guidance is often concentrated at the undergraduate level (31), while high-school students face time constraints and limited opportunities for social/clinical exposure, reinforcing the need to advance guidance earlier in the pipeline. Consequently, high school students heavily rely on adults for objective analysis of pros and cons (32). This dynamic could explain why in our study students with strong family support had a much higher intention to choose pediatrics, which aligns with prior research highlighting the significant influence of familial opinions and teacher guidance on major decision-making (32–34). Furthermore, we also observed a correlation between the presence of medical professionals within the family and students’ inclination towards pediatric specialties, possibly due to parental careers sparking their interest in medicine (35). However, it is imperative to conduct further investigation into the influence of familial backgrounds on students’ career choices and the development of healthcare human resources. This issue necessitates additional research.

We observed higher willingness among humanities-track students than among science-track students (8.5% vs. 6.0%). Two non-mutually exclusive explanations may apply. First, under the reformed gaokao in some provinces, certain humanities-oriented subject combinations may still permit eligibility for medical majors, potentially broadening the pool of interested students. Second, some humanities-track students may express aspirational interest without fully internalizing science prerequisites and training requirements, consistent with prior observations that early-stage intentions can reflect information gaps about major-specific pathways. Accordingly, we treat this finding as secondary yet noteworthy and urge cautious interpretation given policy heterogeneity across provinces and the self-reported nature of track selection. The location gradient—higher willingness in smaller cities/towns and lower willingness in top-tier metros—may reflect opportunity sorting (broader alternatives/competition in metropolitan areas) versus perceived local need and attainability in smaller locales. Given the small Tier-1/2 samples, these estimates warrant cautious interpretation.

The results of this research suggest that high school students with a keen interest in pursuing a career as pediatricians are more inclined to apply for related opportunities. This pattern reinforces that genuine passion for medicine is a primary driver of interest in pediatrics. According to career theory, the satisfaction and longevity of one’s chosen profession are contingent upon their individual interests and values (36). Personal interest has consistently served as a pivotal determinant in the selection of one’s professional path (20, 37, 38). When the alignment of professional choice and personal interests is achieved, it can bolster professional engagement and identity, while also unlocking one’s unique personality traits and talents (34). Practically, early interest-building interventions—such as school-based science and health outreach, structured shadowing/volunteering opportunities, exposure to pediatric role models, and clear communication about pediatric training, workload, and career trajectories—may help spark and sustain motivation among prospective applicants.

Within our study, we demonstrated that a higher proportion of senior high school students who possess an enhanced comprehension of pediatrics or careers through pandemic publicity or personal experience exhibit a stronger inclination to enroll. These patterns suggest that while pandemic-related anxiety is associated with a small uptick in interest, heightened recognition of pediatrics after COVID-19 is a much stronger correlate of willingness to choose pediatrics. A plausible interpretation is that firsthand exposure to pandemic narratives and needs increased students’ awareness, perceived social value, and sense of calling toward pediatric care, beyond general concerns about the future. These patterns suggest that salient public-health events—through hero narratives, prosocial framing, and heightened awareness of societal need—can nudge career intentions, particularly when paired with accurate, concrete information about pediatric pathways (29). Because such effects may be short-lived without reinforcement, we recommend timely, structured outreach (e.g., school seminars by pediatricians, hospital open days, authentic media storytelling, and student shadowing/volunteering) to convert momentary inspiration into sustained, informed interest in pediatrics.

This finding suggests that reality-aligned expectations—formed after a concise, factual introduction to pediatric training and work—are positively associated with willingness to choose pediatrics, underscoring the value of accurate, concrete information in recruitment messaging. Moreover, 80.5% of students consider “job stability and future career benefits” to be highly important factors influencing their decision. Interestingly, while the vast majority value job stability/benefits, those placing the highest emphasis were somewhat less likely to choose pediatrics, suggesting that students drawn to pediatrics may be motivated by intrinsic interest or a sense of calling, whereas those most focused on stability may gravitate toward fields they perceive as more secure or lucrative.

Our results show that students who perceive better benefits and career prospects in pediatrics are more likely to express strong intention to pursue the field (Tables 2, 3). Conversely, perceived low income and poor advancement opportunities appear to dampen interest, mirroring the broader literature on job satisfaction and turnover in public hospitals (39). These patterns suggest that policy levers on the employer side—notably raising compensation, improving staffing ratios to reduce workload and burnout, and establishing clear, credible promotion pathways for pediatricians—could improve retention of current staff and also recruitment by making pediatrics more attractive to prospective entrants. In parallel, formal recognition of pediatricians’ technical value (e.g., performance-based pay components, recognition of cognitive/communication-intensive skills, and transparent criteria for advancement) would align incentives with the specialty’s actual demands. Taken together, making pediatrics more rewarding both financially and professionally is likely to have dual benefits—stabilizing the existing workforce and strengthening the pipeline by increasing students’ willingness to choose pediatrics.

Although our study utilized a sample of high school students from four provinces in China with varying levels of economic development, the results may not be fully representative of all regions in China. We did not power or pre-specify province-level comparisons (e.g., Guangdong vs. Henan), so potential geographic heterogeneity warrants confirmation in larger, stratified, nationally representative studies. The study uses self-reported student data, which may be biased due to social desirability, leading to responses that reflect what students think researchers want, rather than their true thoughts. Several variables rely on subjective perceptions that may be influenced by media exposure or local policy environments, and residual confounding may remain despite multivariable adjustment. Some subgroups are relatively small (e.g., Tier-1/2 origins, medical-parent families), so estimates for those categories should be interpreted cautiously. Additionally, the study’s time constraints did not allow for tracking whether the subjects ultimately chose pediatrics as their profession. Motivations for choosing pediatrics may vary across different settings, and the findings may not be fully applicable to other countries or cultural contexts, but they could still serve as a reference for developing countries facing doctor shortages. Future research using prospective designs, broader regional coverage, and mixed methods (including qualitative interviews and intervention studies on counseling/role-model exposure) will help clarify mechanisms and inform targeted recruitment strategies.

## 5 Conclusion

In a large, multi-province sample of high-school students, only ∼6% expressed a strong intention to pursue pediatrics, highlighting a significant pipeline gap. Our findings point to several actionable priorities:

### 5.1 Targeted recruitment

Tailor outreach to address gender differences (e.g., highlight career impact, advancement stories, and technical skill recognition to better engage male students while sustaining female interest). Engage families and communities—given the strong effect of family support—through parent-inclusive events (school talks by pediatricians, hospital open days, mentoring).

### 5.2 Education and guidance

Students with medical professional parents were more inclined toward pediatrics—likely due to firsthand exposure. To narrow information gaps for others, parent- and teacher-inclusive outreach (hospital open days, school talks by pediatricians, mentoring/shadowing opportunities, and clear pathway maps outlining training time, competencies, and career prospects) can sustain interest even as students gain a more realistic understanding of workload, remuneration, and the doctor–patient climate. These observations support earlier, targeted career education at the high-school stage: clarify the nature of pediatric work, training length, and career prospects; expand firsthand exposure (shadowing/volunteering, skills workshops); and leverage university outreach/new media that were associated with higher interest in our data, involving not only students but also parents and teachers, to align interests, expectations, and opportunities.

### 5.3 Systemic improvements

Strengthen the public image of pediatrics and address doctor–patient relationship concerns through balanced media and community outreach. In parallel, policy and employer-side reforms—including raising income levels, improving staffing ratios to reduce workload, and establishing clear promotion pathways—can enhance job satisfaction, professional identity, and retention while making the field more attractive to new entrants.

### 5.4 Broader implications and ongoing evaluation

Because societal and educational contexts evolve, continuous research and monitoring are needed to track students’ aspirations and the changing needs of pediatric education. Insights from such evaluations can guide educational institutions and health systems in developing targeted, evidence-based strategies to attract and nurture future pediatricians—thereby helping to address pediatrician shortages and strengthen child health services, including in resource-constrained and developing settings.

## Data Availability

The original contributions presented in this study are included in this article/Supplementary material, further inquiries can be directed to the corresponding authors.

## References

[1] Mileder LP. Learning from pediatrics to secure medical education for the future. *Acad Med*. (2018) 93:523–4. 10.1097/ACM.0000000000002138 29341998

[2] National Bureau of Statistics of China. *What census data can tell us about children in China.* Beijing: National Bureau of Statistics of China (2013)

[3] Li Q Li X Zhang Q Zhang Y Liu L Cheng X A cross-sectional nationwide study on accessibility and availability of neonatal care resources in hospitals of China: current situation, mortality and regional differences: neonatal care resources and Newborn Mortality in China. *Lancet Reg Health West Pac*. (2021) 14:100212. 10.1016/j.lanwpc.2021.100212 34528000 PMC8358159

[4] Feng X Wang Y Wei L Meng K. How to become an excellent pediatric resident: a qualitative comparative study from China. *BMC Health Serv Res*. (2023) 23:53. 10.1186/s12913-023-09038-x 36653822 PMC9850579

[5] Hou J Michaud C Li Z Dong Z Sun B Zhang J Transformation of the education of health professionals in China: progress and challenges. *Lancet*. (2014) 384:819–27. 10.1016/S0140-6736(14)61307-6 25176552

[6] Zhuo LTH Wu Q. Perceived doctor-patient relationship and turnover intention among pediatricians: the mediating role of job satisfaction. *China Health Policy Res.* (2021) 14:43–8. 10.3969/j.issn.1674-2982.2021.11.006

[7] Rackley S Bostwick JM. The pediatric surgeon-patient relationship. *Semin Pediatr Surg*. (2013) 22:124–8. 10.1053/j.sempedsurg.2013.04.002 23870204

[8] Vaishnav BS Vaishnav SB Chotaliya M Bathwar D Nimbalkar S. Cognitive style assessment among medical students: a step towards achieving meta-cognitive integration in medical education. *Natl Med J India*. (2019) 32:235–8. 10.4103/0970-258X.291298 32769247

[9] Wang X. Reasons for the Lack of Pediatricians in China and Countermeasures. (2016). p. 312–3. 10.3760/cma.j.issn.1000-6672.2016.04.021

[10] Chen S Chen H Ling H Gu X. An online career intervention for promoting Chinese high school students’. *Career Readiness. Front Psychol*. (2021) 12:815076. 10.3389/fpsyg.2021.815076 35082735 PMC8784529

[11] Kusurkar R Kruitwagen C ten Cate O Croiset G. Effects of age, gender and educational background on strength of motivation for medical school. *Adv Health Sci Educ Theory Pract*. (2010) 15:303–13. 10.1007/s10459-009-9198-7 19774476 PMC2940046

[12] Deng S Yang N Li S Wang W Yan H Li H. Doctors’ job satisfaction and its relationships with doctor-patient relationship and work-family conflict in China: a structural equation modeling. *Inquiry*. (2018) 55:46958018790831. 10.1177/0046958018790831 30371128 PMC6207965

[13] Zhang H Liu X Penn-Kekana L Ronsmans CA. systematic review of the profile and density of the maternal and child health workforce in China. *Hum Resour Health*. (2021) 19:125. 10.1186/s12960-021-00662-4 34627289 PMC8501553

[14] Deng W Feng Z Yao X Yang T Jiang J Wang B Occupational identity, job satisfaction and their effects on turnover intention among Chinese Paediatricians: a cross-sectional study. *BMC Health Serv Res*. (2021) 21:6. 10.1186/s12913-020-05991-z 33397391 PMC7780641

[15] Shi M Shi Y Zhao Z Zhai X Fan X. The effect of family structure on physical activity levels among children and adolescents in Western China in the era of COVID-19. *BMC Public Health*. (2022) 22:2072. 10.1186/s12889-022-14432-x 36376883 PMC9660204

[16] Ma S Xu X Trigo V Ramalho NJ. Doctor-patient relationships (DPR) in China. *J Health Organ Manag*. (2017) 31:110–24. 10.1108/JHOM-09-2016-0165 28260407

[17] Silva CA Trindade VC Abel RCD Silva MO Santos JFV Koch VHK Pediatricians after residency: a survey of personal/professional data and issues. *Rev Paul Pediatr.* (2021) 39:e2019190. 10.1590/1984-0462/2021/39/2019190 32756760 PMC7401498

[18] Wen T Zhang Y Wang X Tang G. Factors influencing turnover intention among primary care doctors: a cross-sectional study in Chongqing, China. *Hum Resour Health*. (2018) 16:10. 10.1186/s12960-018-0274-z 29433519 PMC5809822

[19] Goetz K Musselmann B Szecsenyi J Joos S. The influence of workload and health behavior on job satisfaction of general practitioners. *Fam Med.* (2013) 45:95–101. 23378076

[20] Waterval D Frambach JM Scott SM Driessen EW Scherpbier AJJA. Crossborder curriculum partnerships: medical students’ experiences on critical aspects. *BMC Med Educ*. (2018) 18:129. 10.1186/s12909-018-1239-6 29879976 PMC5992638

[21] Hu L Wu H Zhou W Shen J Qiu W Zhang R Positive impact of COVID-19 on career choice in pediatric medical students: a longitudinal study. *Transl Pediatr*. (2020) 9:243–52. 10.21037/tp-20-100 32775243 PMC7347769

[22] Laurence D Görlich Y Simmenroth A. How do applicants, students and physicians think about the feminisation of medicine? - a questionnaire-survey. *BMC Med Educ*. (2020) 20:48. 10.1186/s12909-020-1959-2 32046693 PMC7014700

[23] Lin S Shi H Ruan J. A comparative study of occupation satisfaction and career development between pediatric practitioners and non-pediatric practitioners: a survey of pediatric graduates. *J Wenzhou Med Univ.* (2023) 53:161–73.

[24] Roter DL Hall JA Aoki Y. Physician gender effects in medical communication: a meta-analytic review. *JAMA*. (2002) 288:756–64. 10.1001/jama.288.6.756 12169083

[25] Valantine H Sandborg CI. Changing the culture of academic medicine to eliminate the gender leadership gap: 50/50 by 2020. *Acad Med*. (2013) 88:1411–3. 10.1097/ACM.0b013e3182a34952 23969359 PMC3785938

[26] Pololi LH Civian JT Brennan RT Dottolo AL Krupat E. Experiencing the culture of academic medicine: gender matters, a national study. *J Gen Intern Med*. (2013) 28:201–7. 10.1007/s11606-012-2207-1 22936291 PMC3614142

[27] Abramson EL Weiss P Naifeh M Stevenson MD Duncan JG Rama JA Scholarly activity during pediatric fellowship. *Pediatrics*. (2021) 147:e2020013953. 10.1542/peds.2020-013953 33262266

[28] Hang Y Huang L Zhou X Zhang X Dong X Fang J Characteristics and workload of pediatricians in China. *Pediatrics.* (2019) 144:e20183532. 10.1542/peds.2018-3532 31253739

[29] Kim JH Shin HS. Effects of self-reflection-focused career course on career search efficacy, career maturity, and career adaptability in nursing students: a mixed methods study. *J Prof Nurs*. (2020) 36:395–403. 10.1016/j.profnurs.2020.03.003 33039075

[30] Lee SY Lee MJ Lee SH. Analysis on Students’ Career Preparation in One Korean Medical School: based on the relationship and trend between career maturity and specialty indecision. *J Korean Med Sci*. (2022) 37:e277. 10.3346/jkms.2022.37.e277 36163476 PMC9512679

[31] Greenberg RB Ziegler CH Borges NJ Elam CL Stratton TD Woods S. Medical student interest in academic medical careers: a multi-institutional study. *Perspect Med Educ*. (2013) 2:298–316. 10.1007/s40037-013-0051-6 23670688 PMC3824757

[32] Popper-Giveon A Keshet Y. “It’s Every Family’s Dream”: choice of a medical career among the arab minority in Israel. *J Immigr Minor Health*. (2016) 18:1148–58. 10.1007/s10903-015-0252-7 26175137

[33] Simmenroth-Nayda A Görlich Y. Medical school admission test: advantages for students whose parents are medical doctors? *BMC Med Educ.* (2015) 15:81. 10.1186/s12909-015-0354-x 25898946 PMC4409754

[34] Wouters A Croiset G Isik U Kusurkar RA. Motivation of Dutch high school students from various backgrounds for applying to study medicine: a qualitative study. *BMJ Open*. (2017) 7:e014779. 10.1136/bmjopen-2016-014779 28576893 PMC5623448

[35] Zhang Y Huang L Zhou X Zhang X Ke Z Wang Z Characteristics and workload of pediatricians in China. *Pediatrics*. (2019) 144:e20183532. 10.1542/peds.2018-3532 31253739

[36] Nye CD Su R Rounds J Drasgow F. Vocational interests and performance: a quantitative summary of over 60 years of research. *Perspect Psychol Sci*. (2012) 7:384–403. 10.1177/1745691612449021 26168474

[37] Goel S Angeli F Dhirar N Singla N Ruwaard D. What motivates medical students to select medical studies: a systematic literature review. *BMC Med Educ*. (2018) 18:16. 10.1186/s12909-018-1123-4 29343262 PMC5772649

[38] Kim KJ Hwang JY Kwon BS. Differences in medical students’ academic interest and performance across career choice motivations. *Int J Med Educ*. (2016) 7:52–5. 10.5116/ijme.56a7.5124 26878567 PMC4764248

[39] Moreno-Jiménez B Gálvez-Herrer M Rodríguez-Carvajal R Sanz Vergel AI. A study of physicians’ intention to quit: the role of burnout, commitment and difficult doctor-patient interactions. *Psicothema.* (2012) 24: 263–70. 22420355

