# Supplementary material for: Why choose pediatrics? A survey on factors influencing Chinese high school students’ choice of pediatrics major
Source: Front Med (Lausanne). 2026 Jan 21;12:1646958. doi: 10.3389/fmed.2025.1646958 (PMC12867865; doi:10.3389/fmed.2025.1646958)
Supplement: Supplementary file 1 [file Table_1.docx]

**Questionnaire of high school students applying for medical majors in the post-epidemic period**

**Dear students, hello! We are a team from the Medical School of Shanghai Jiao Tong University. After you have accepted the popularization of various medical specialties and industry-related information, in order to further understand your willingness to apply for medical majors, I hope you can fill in this questionnaire accurately and truthfully. This questionnaire is an anonymous survey, and the results will not be used in other ways than for research. Thank you for your help, and I hope that our introduction of medical majors is helpful to you.**

(Answer description: There are four types of questions, respectively, single choice, multiple choice, fill in the blank, form questions. Among them, multiple choice and filling in the blanks are marked, and those without special instructions are single choice.

Answer method: multiple-choice questions: fill in the option serial number in parentheses;Fill in the blanks: fill in the answer on the horizontal line according to the question requirements;Table question: tick the corresponding grid "" according to your opinion. Note: can only check one option for each line.)

**Basic information class**

**1, your gender: ( )**

A, male, B, and female

**2, your grade: ( )**

A, Grade one B, Grade two C, Grade three D, repeat reading

**3. Your grade is as follows: ( )**

A, Top 5% B, Top 30% C, Medium (top 30% to top 70%) D, last 30% E ,last 5%

**4. Your course selection is: ( )**

A, liberal arts (partial liberal arts) B, science (partial science)

**5. Please write down about your province: [gap filling]**

**6. Your frequent place of residence is: ( )**

A, first-tier cities B, second-tier cities C, third-tier cities D, town E, rural areas

**7. Are your parents medical workers?()**

A, only the father is a medical worker B, only the mother is A medical worker

C, parents are medical workers D, parents are not medical workers

**8. Your family's monthly income is about: ( )**

A, less than 300 dollars B, 300 ~700 dollars C, 700 ~ 1400 dollars

D, 1400 ~2800 dollars E,2800 dollars above

**9. Have you and your relatives and friends ever suffered from a serious** disease or a disease that cannot be cured by drugs? ( ), A,Yes, B,No

**10. Do your family members or familiar people have the following occupations?( )** [Multiple choice]

A, clinical or pediatrician B, nurse C, physician or technician

D, basic medical researchers

E, public health and preventive medicine-related occupations

F, pharmacy-related occupations G, none

**Epidemic related**

**11. Since the outbreak of the epidemic, the highest risk level in your area is: ( )** A, high risk B, medium risk C, low risk

**12. The impact of the epidemic on your current life is follows: [Please fill in any integer in 1~5]**

○ 1 (very low impact) ○ 2 ○ 3 ○ 4 ○ 5 (great impact)

**13. Have you ever experienced a diagnosis or 14 days of independent medical isolation?( )**

A, Yes, I was in confirmed case B, Yes, I was in quarantinC, no, but the people I know have experienced D, no, I and the people around me have not experienced

1. **Have any family members become the frontline medical** workers fighting against the epidemic?(),
2. A, Yes, B,No

**15. During the epidemic, I have felt panic, anxiety, and depression: [Please fill in 1~5]**

○ 1 (totally inconsistent) ○ 2 ○ 3 ○ 4 ○ 5 (fully compliant)

**Professional tendencies and professional values**

**16, please choose the most consistent three descriptions of your personality according to your self-knowledge:** ( ) [multiple choice]

A,social type (like to communicate with people, care about social issues, value social obligations and social morality)

B,enterprise type (the pursuit of power, authority and material wealth, do things with A strong purpose)

C,Regular type (respect for authority and regulations, cautious and conservative, lack of creativity, self-sacrifice)

D. Practical (willing to use tools, strong hands-on ability), lack of social skills, like to work independently

E, research type (strong abstract thinking ability, like logical analysis and reasoning, but unwilling to do it)

F, artistic (creative, idealized, not practical, with artistic talent and personality)

**17, please choose your preferred university major type: ( )**

1. Experimental research (scientific experiment, observation and analysis, logical reasoning, rational thinking)
2. Business management (finance and business, organization and management leaders)

C. Mechanical operation type (operating tools, machinery, equipment, follow the working procedure to manufacture the product)

D, natural exploration (focus on nature, universe planet, explore life phenomena, natural laws) E, affairs planning (systematic, organized, planning, attention to details, attention to affairs)

F. Social assistance (ready to help others, serve the society, social communication)

G, art and literature type (literature and art innovation, rich emotion, the pursuit of aesthetic feeling)

**18 Who do you think has the biggest influence on you in the choice of college major**

A, decide by themselves B, parents C, teacher D, related professionals

**19 Do you have a clear professional orientation or career plan?( )** A, Yes, B, and No

**20 The future professional high social status, high administrative level, for you?( )**

A, very important B, more important C, generally important D, less important E, very not important

**21The future career is in line with the interests and hobbies, can play the talent, professional counterpart, for you?( )**

A, very important B, more important C, generally important D, less important E, very not important

**22 The future career job stability, good welfare, full insurance, for you?( )**

A, very important B, more important C, generally important D, less important E, very not important

**23 What do you think is the nature of the following occupations?[Only one corresponding option can be checked per line]**

|  | profit is all that matters | service industry | Technical work | Help others |
| --- | --- | --- | --- | --- |
| Clinical physician, or a pediatrician |  |  |  |  |
| nurse |  |  |  |  |
| Hospital auxiliary department physician or technician |  |  |  |  |
| Basic medical researchers |  |  |  |  |
| Pharmacy researcher |  |  |  |  |

**The intention to enter the examination is related**

**24 How do you primarily learn about university majors? ( )**

A, traditional media (newspapers, magazines, etc.) B, new media (network, app, etc.) C, university publicity

D, people around experience / talk about E, do not understand

**25. How do you mainly learn about the following medical majors and their related professions?[Only one corresponding option can be checked per line]**

|  | legacy media  (Newspapers, magazines, etc.) | new media  (Network, app, etc.) | University propaganda | Experiences / talk of the people around you | do not understand |
| --- | --- | --- | --- | --- | --- |
| clinical medicine |  |  |  |  |  |
| pediatrics |  |  |  |  |  |
| nurse |  |  |  |  |  |
| pharmacy |  |  |  |  |  |
| public health |  |  |  |  |  |
| preclinical medicine |  |  |  |  |  |
| medical image |  |  |  |  |  |
| Medical laboratory technology |  |  |  |  |  |

**26. How often you contact the relevant information about the following medical majors is: [You can only check one corresponding option for each line]**

|  | Never contact | Very low | lower | higher | polar altitude |
| --- | --- | --- | --- | --- | --- |
| clinical medicine |  |  |  |  |  |
| pediatrics |  |  |  |  |  |
| nurse |  |  |  |  |  |
| pharmacy |  |  |  |  |  |
| public health |  |  |  |  |  |
| preclinical medicine |  |  |  |  |  |
| medical image |  |  |  |  |  |
| Medical laboratory technology |  |  |  |  |  |

**27. Compared with the professional profile, is your previous knowledge of the following medical majors consistent?[Check one option per line]**

|  | be completely in conformity with | Compare in line with | Relatively general | The comparison is not consistent | It doesn't fit |
| --- | --- | --- | --- | --- | --- |
| clinical medicine |  |  |  |  |  |
| paediatrics |  |  |  |  |  |
| nurse |  |  |  |  |  |
| pharmacy |  |  |  |  |  |
| public health |  |  |  |  |  |
| preclinical medicine |  |  |  |  |  |
| medical image |  |  |  |  |  |
| Medical laboratory technology |  |  |  |  |  |

**28 Have you ever participated in medical-related practicalactivities or popular science activities?()**A, Yes B, No

**29. Are you willing to participate in medical-related practical activities or popular science activities?()** A, Yes, B, and No

**30 If you popularize more medical knowledge, what way would you prefer most?( )**

A, Traditional media (newspapers, magazines, etc.) B, new media (network, public account, small video, etc.) C, lecture

**31. Do you want to apply for the medical major?( )**

**[Single choice: choose "Yes" to complete 32 questions, do not complete 33 questions; choose "No" to complete 33 questions, do not complete 32 questions] A, Yes B, no**

**32. Has the outbreak of COVID-19 affected your application for** a medical major?() A, no impact B, positive impact C, negative impact

**33 Has the outbreak of COVID-19 erased your willingness to study medicine?(),**

A, Yes, B, and No

**34. What attitude do your family members or familiar people hold towards applying for the following medical majors?[Check one corresponding option for each line]**

|  | set oneself against | There have been objections | Relative neutral | Support has been raised | scream for |
| --- | --- | --- | --- | --- | --- |
| clinical medicine |  |  |  |  |  |
| pediatrics |  |  |  |  |  |
| nurse |  |  |  |  |  |
| pharmacy |  |  |  |  |  |
| public health |  |  |  |  |  |
| preclinical medicine |  |  |  |  |  |
| medical image |  |  |  |  |  |
| Medical laboratory technology |  |  |  |  |  |

**35. Your current willingness to apply for the following medical majors is respectively: [You can only check one corresponding option in each line]**

|  | Very weak | relatively weak | same as | stronger | pole-strength |
| --- | --- | --- | --- | --- | --- |
| clinical medicine |  |  |  |  |  |
| pediatrics |  |  |  |  |  |
| nurse |  |  |  |  |  |
| pharmacy |  |  |  |  |  |
| public health |  |  |  |  |  |
| preclinical medicine |  |  |  |  |  |
| medical image |  |  |  |  |  |
| Medical laboratory technology |  |  |  |  |  |

**36. After the outbreak of the epidemic, have you increased your active understanding of the following majors?[Only one corresponding option per column]**

|  | clinical medicine | paediatrics | nurse | pharmacy | public health | preclinical medicine | medical image | Medical laboratory technology |
| --- | --- | --- | --- | --- | --- | --- | --- | --- |
| yes |  |  |  |  |  |  |  |  |
| deny |  |  |  |  |  |  |  |  |

**Views of the doctor-patient relationship**

**37 What is the overall satisfaction of you and the people around you with your medical experience?()**

A, very satisfied B, more satisfied C, relatively generalD, relatively Unsatisfied E, very dissatisfied

**38 Your cognition of the current tension degree of the doctor-patient relationship is: ( )**

A, very harmonious, B, relatively harmonious, C, relatively general, D, relatively nervous, E, very nervous

**39 Do you think the doctor-patient relationship in China in the next few years will be: ( )**

A, improved B, basically unchanged C, deteriorated

**Clinical medicine**

**40 Your understanding of the length of schooling of clinical medicine major (especially the long length of schooling) is: ( ) (This length of schooling refers to the number of years of study, if the gegneral major undergraduate length is four years)**

A, very understanding B, more understanding C, relatively general

D. Unfamiliar with E, or completely ignorant

**41 What do you think of the future of the doctor industry?( )**

A, very good B, better C, general D, relatively poor E, very poor

**42 What do you think of the social responsibility of doctors?( )**

A, very large B, relatively large C, general D, relatively small E, very small

**43 What do you think of the social status of doctors today?( )**

A, very high B, relatively high C, general D, low E, very low

**44 All kinds of media, articles in shaping the image of doctors,which image makes you more impressive?()**

A, positive image B, negative image

**Pediatric major**

**45 Your understanding of the following questions: [each line can only check one corresponding option]**

|  | Very low (poor) | Relatively Low (Poor) | same as | Relatively High (good) | Very high (good) |
| --- | --- | --- | --- | --- | --- |
| Degree of interest in becoming a pediatrician |  |  |  |  |  |
| Knowledge of the future direction of pediatric graduates |  |  |  |  |  |
| Knowledge of time cost for pediatricians in Grade A hospitals (8 years) |  |  |  |  |  |
| The current employment situation of pediatricians |  |  |  |  |  |
| Current medical work environment for pediatricians |  |  |  |  |  |
| Current work pressure of pediatricians |  |  |  |  |  |
| The current social status of pediatricians |  |  |  |  |  |
| The current sense of professional accomplishment of pediatricians |  |  |  |  |  |
| The current salary and treatment of pediatricians |  |  |  |  |  |
| The overall image of pediatricians |  |  |  |  |  |

**46 Do you know of the existence of private pediatric clinics?( )**

A, Yes, B, and No

**47 What kind of hospital do you prefer to work in?( )**

A, public hospital B, private practice

**48. Compared with public hospitals, you know about pediatricians in private clinics as follows: [You can only check one corresponding option for each line]**

|  | Worse (low) | almost | Better (high) |
| --- | --- | --- | --- |
| Social recognition |  |  |  |
| Service image |  |  |  |
| Pay the income ratio |  |  |  |
