# Supplementary material for: Why choose pediatrics? A survey on factors influencing Chinese high school students’ choice of pediatrics major
Source: Front Med (Lausanne). 2026 Jan 21;12:1646958. doi: 10.3389/fmed.2025.1646958 (PMC12867865; doi:10.3389/fmed.2025.1646958)
Supplement: Supplementary file 2 [file Table_2.docx]

**Ethics Approval Document of Scientific Research Project in Medical Ethics Committee of Shanghai First People's Hospital**

| **Approval number** 2019KY058 | | **Review date** 2019.10.23 | | **Item Number** 2019 Section 058 | |
| --- | --- | --- | --- | --- | --- |
| **Project name** | Investigation on Contemporary Students' Willingness to Practice Medicine | | | | |
| **Project source** | Self-funded | | | | |
| **Main researcher** | Deng Guoying | | | | |
| **Research unit** | Shanghai First People's Hospital | | **Department** | | Trauma center |
| **The review documents (including version numbers) are as follows：**  (1) Research plan  (2) informed consent application  (3) Resume of the main researcher | | | | | |
| **1、Review method**   - Meeting review - Quick review - Emergency meeting review   **2、Review result**  Consent  3、The research will be subject to continuous review by the ethics committee during the process   - Yes - No   The review frequency is once every **12** months from the date of approval of the study.  The ethics committee has the right to change the frequency of continuous review based on actual progress.  4、The approval period is **36** months, Until **October 23, 2022.**  Hospital Ethics Committee (stamp):  Date: October 23,2019 | | | | | |

Address: 100 Haining Road, Shanghai (200080) Tel: 63240090
